# Exploring the focus of prenatal information offered to pregnant mothers regarding newborn care in rural Uganda

**DOI:** 10.1186/1471-2393-13-176

**Published:** 2013-09-16

**Authors:** Mangwi Richard Ayiasi, Kathleen Van Royen, Roosmarijn Verstraeten, Lynn Atuyambe, Bart Criel, Christopher Orach Garimoi, Patrick Kolsteren

**Affiliations:** 1 Mulago hospital complex, Makerere University School of Public Health, P.O Box 7072, Kampala, Uganda; 2 Faculty of Bioscience Engineering Coupure, Ghent University, Links 653, 9000 Ghent, Belgium; 3 Institute of Tropical Medicine, Nationalestraat 155, B-2000 Antwerp, Belgium; 4 Department of Communication Sciences, University of Antwerp, Sint-Jacobstraat 2, 2000 Antwerp, Belgium

## Abstract

**Background:**

Neonatal death accounts for one fifth of all under-five mortality in Uganda. Suboptimal newborn care practices resulting from hypothermia, poor hygiene and delayed initiation of breastfeeding are leading predisposing factors. Evidence suggests focused educational prenatal care messages to mitigate these problems. However, there is a paucity of data on the interaction between the service provider and the prenatal service user. This study aims to understand the scope of educational information and current practices on newborn care from the perspectives of prenatal mothers and health workers.

**Methods:**

A qualitative descriptive methodology was used. In-depth interviews were conducted with lactating mothers (n = 31) of babies younger than five months old across Masindi in western Uganda. Additional interviews with health workers (n = 17) and their employers or trainers (n = 5) were conducted to strengthen our findings. Data were audio-taped and transcribed verbatim. A thematic content analysis was performed using NVivo 8.

**Results:**

Vertical programmes received more attention than education for newborn care during prenatal sessions. In addition, attitudinal and communication problems existed among health workers thereby largely ignoring the fundamental principles of patient autonomy and patient-centred care. The current newborn care practices were largely influenced by relatives’ cultural beliefs rather than by information provided during prenatal sessions. There is a variation in the training curriculum for health workers deployed to offer recommended prenatal and immediate newborn care in the different tiers of health care.

**Conclusions:**

Findings revealed serious deficiencies in prenatal care organisations in Masindi. Pregnant mothers remain inadequately prepared for childbirth and newborn care, despite their initiative to follow prenatal sessions. These findings call for realignment of prenatal care by integrating education on newborn care practices into routine antenatal care services and be based on principles of patient-centred care.

## Background

Of all 4.2 million deaths in children under five years in sub-Saharan Africa, 29% are neonates [1]. In Uganda, 22.5% of under-five mortality is attributable to neonatal mortality [2]. The majority of neonatal deaths occur within the first week of life, as a result of suboptimal care during the prenatal and immediate postnatal period [3,4]. The predisposing conditions for neonatal morbidity and mortality include poor thermal care, poor hygiene practices, especially regarding care for the cord, and delayed initiation of breastfeeding [5]. Most of these conditions are preventable by basic inexpensive promotional interventions offered during the prenatal period [6,7].

Prenatal care provides an important entry point for pregnant women into the health care system [8] and offers a unique opportunity to organise the necessary services for pregnant women in order to ensure a healthy pregnancy, safe delivery and a healthy mother-baby pair [5]. The World Health Organisation (WHO) recommends a series of prenatal services, based on the four-visit model, to be offered to pregnant women depending on gestational age and individual needs, i.e. focused antenatal care (FANC) [8].

The concept of FANC targets the unique challenges of each pregnant woman [9] and includes health promotion and disease prevention; clinical examinations and history taking for the early detection and treatment of complications and existing diseases; birth preparedness and complication readiness [5,10,11]. It has been argued that FANC provided by skilled health staff can have a positive impact on institutional delivery [12,13], improve newborn care practices and therefore contribute to overall child mortality reduction [14,15].

Implementation of FANC in sub-Saharan Africa for over a decade resulted in prominent levels of the number of women making at least one prenatal visit (80-90% or more) [16-19]. In Uganda, FANC attendance rates of up to 96% have been reported [20]. However high FANC attendance has not improved newborn care practices, especially in developing countries like Uganda where neonatal mortality is estimated at 26 deaths per thousand live births. This makes the attainment of the fourth Millennium Development Goal elusive [21].

In Uganda, available data on the prevalence and determinants of potentially harmful newborn care practices [22,23] indicate that care practices are deeply rooted in culture and beliefs. The “Uganda Newborn Survival Study” in the Iganga-Mayuge Demographic Surveillance Sites (DSS), [22], exploring newborn care practices in communities and the formal health care system, reported major issues: low adherence to newborn care practices in the homes especially among the poorer segments of communities; delay in seeking for care which has been associated with late recognition of symptoms and homecare practices that are potentially harmful for the newborn. In addition, these studies recommended interventions early in pregnancy emphasising FANC in order to affect behaviour change. However, they remain silent about how FANC can be implemented to affect the desired newborn care practices.

Studies conducted to evaluate FANC implementation elsewhere in Africa usually assessed its outcomes in terms of maternal and neonatal morbidity and mortality or acceptability of the reduced visits among users and providers [24] ignoring the crucial interaction between the health care provider and its users. There is scant data on the scope of prenatal care offered to pregnant women.

Because a successful prenatal education programme should be based on the current practices of those to whom the intervention is targeted, we set out to gather information on the current practices of newborn care and the scope of information offered during prenatal education. In addition, we aimed to explain the reasons for suboptimal newborn care practices despite the high prenatal attendance in two rural districts of Uganda by exploring perceptions of both mothers and health workers, including their tutors at the training school and employers at the district.

## Methods

### Study setting

This study was conducted in the rural districts of Masindi and Kiryandongo [later in the text referred to as Masindi district: this study was designed before the Masindi district in July 2011 was divided into Masindi and Kiryandongo; we will therefore maintain the original name of Masindi] in November - December 2011. Masindi is located about 214 km Northwest of Kampala and had a population of 603,000 in 2010, which was predominantly rural with only 5.4% being urban.

Health services in Uganda are decentralised to district local governments. Recruitment of health workers is conducted by the District Service Commission on behalf of the district local government, while staff deployment is done by the Chief Administrative Officer. Health facilities in the district health system are organized into a four-tier system: health centre of levels 2, 3, 4 and hospital. All four levels of care are mandated to offer antenatal care and delivery services. In Masindi, there are two hospitals, one health centre of level 4, 10 health centres of level 3 and 21 health centres of level 2. Two broad categories of nursing cadres are deployed in the district health system- midwives and general nurses. They can either be enrolled nurses/midwives with basic skills or registered nurses/midwives with advanced skills. During training midwives are given basic orientation in general nursing care, likewise, general nurses are given basic orientation in midwifery. This training is meant to develop a polyvalent health worker capable of offering maternal, child and newborn health in addition to general nursing care. Guidelines for health worker establishment, formulated by the Ministry of Health (MOH) in collaboration with the Ministry of Public Service, require that health centres of levels 2 and 3 should employ enrolled nurse/midwife with basic nursing/midwifery training, while the registered nurse/midwife with advanced skill is employed at health centres of level 4 and hospital.

### Sample and recruitment

We conducted 32 in-depth interviews with mothers to identify their perspectives on the scope of information received during prenatal sessions and their current newborn care practices. To include opinions and experiences from different age categories, both adult and adolescent women were selected from each of the 16 sampled parishes in Masindi. Eligible mothers needed to have a child under five months old. Interviews were conducted in their home environment by a social scientist that was familiar with the topic, had a good knowledge of the local dialects commonly spoken in the region and was proficient in qualitative data collection techniques.

Additional interviews with health workers (n = 17) and their trainers or employers (n = 5) were conducted to strengthen our findings and deepen our understanding of the issues identified in the initial interviews on current newborn care practices and the organisation of prenatal sessions in primary health care. Health workers were included in the study if they were a manager of the health care system or a health care provider in the first line. Employers were located at the district level and trainers of health workers were based in the Nurse/Midwifery training school in Mulago where the majority of the nurses and midwives received their training. Interviews for both participant groups were conducted by the principal investigator in English.

Identifiers were provided for each respondent in order to conceal their real identity. Lactating mothers were identified as ‘*adolescent mother’ or ‘adult mother’*, while health workers were identified by their years of experience in service thus *health worker with xx years of experience*. Tutors at the training school were identified as “*tutor training school*” and employers as “*employer at the district”.*

### Ethical considerations

Permission to collect data was obtained from the Higher Degrees Research and Ethics Committee of the School of Public Health, Makerere University and the National Council for Science and Technology in Uganda (number: *HS 1145*). Objectives of the interview were explained to the study participants and written consents secured; a thumb-print was taken in case of illiteracy. Mothers below the consenting age of 18 years were considered as emancipated minors and therefore allowed to provide consent themselves. Confidentiality was assured and the right to opt out at any moment of the interview without any consequences was guaranteed.

### Data collection

Local council leaders were contacted to identify the mothers’ homes. Prior to the interview with the mothers, they provided the following information: age, education level, number of children, a record of attended prenatal visits and place of delivery. A key-word guide was used to ask mothers open-ended questions on their experiences during the prenatal – intra-natal – immediate postnatal period (0–72 hours) for their last pregnancy. If they referred to their previous births this was specifically indicated in the text. The key-word guide was designed to solicit information about their perception on the organisation of prenatal sessions and their current newborn care practices. To better understand care provided during the intra-natal and immediate postnatal period, care practices were explored along three specific thematic areas: i) *cord care* including tying and cutting the cord and application of substance on the umbilical stump; ii) *thermal care* such as drying and wrapping of the baby and bathing; iii) *initiation of breastfeeding* such as how soon breastfeeding was initiated and whether pre-lacteal feeds were offered.

The key-word guide used for the in-depth interviews with the health workers focused on how prenatal care was organised in order to explore implementation and their perceptions on prenatal care. Open-ended questions were followed by more specific probing questions to clarify and extend responses. Interview questions directed to tutors focused on how the curriculum for midwives and nurses was organised and implemented; questions were aimed at understanding how the two categories of nursing cadres were trained to offer information about newborn care. Employers at the district were asked about the recruitment and deployment requirements for the different levels of health care. Both tutors and employers were further solicited on what they considered as the reason for the limited prenatal information offered by nursing staff. Each interview lasted about 60 to 90 minutes and all sessions were audio-recorded and transcribed verbatim.

### Data analysis

The transcribed interviews of the mothers were translated into English. The scripts were carefully read several times and by using a qualitative thematic analysis [25] data were coded into initially predetermined themes and other emerging themes. The codebook was reviewed by a second researcher and any disagreement was settled by consensus. The interviews with mothers were grouped into two age categories (adolescents and adults) to allow for subgroup analyses using matrix coding queries in NVivo 8.

## Results

Thirteen adolescent (14–19 years) and 18 adult (20–45 years) mothers were interviewed. Twenty-six of the respondents were farming their own land, while five were formally employed and earning a monthly salary. Twenty-two mothers attained primary level education, four secondary education and five had no formal education. Mothers visited on average 3.2 times the health care centre for prenatal care; 22 mothers (9 adolescents and 13 adults) delivered at home and 9 (4 adolescents and 5 adults) delivered in a health facility. One interview was excluded from the analysis because of incomplete data.

Four district level managers and 13 peripheral health workers were included in the interviews with health workers. Among the peripheral health workers, 3 were trained as general nurses and 10 received basic midwifery training. Their years of experience ranged from 5 to 30 years. Three of the 17 respondents were men. Three tutors at the training school and two employers at the district level were interviewed.

In this section we present findings along two broad themes: i) the prenatal care organisation and the views of mothers and health workers and ii) the current newborn care practices from the perspective of the mothers. The existing differences between adolescent and adult mothers are only presented where relevant.

### Prenatal care organisation: views of mothers and health workers

#### Delayed, ad hoc and selective implementation of prenatal care

The health care centre attempted to organise prenatal care on a daily basis. As mothers arrived at different times (starting at 7 am onwards) at the health care centre, health workers generally waited for a ‘quorum’ (a reasonable number) before initiating the prenatal session. This made it difficult to decide when to start the session, but usually they did start around 10 to 11 am. In addition, it took long to offer all the services during prenatal visits; the women came early in the morning and could only leave later in the afternoon. Women who arrived late for the session were not offered another chance to receive the information as sessions were only organised on a “collective” basis, and were never a constitutive part of the individual interaction between the patient and the health worker. Some of the health care workers had to say this:

*“But the problem is that the pregnant mothers also come late, some end up coming as late as midday. We try to wait for them up to like about 10–11 a.m., but again we see that this again makes those who come early to wait and really they are complaining. You hear these mothers quarrelling and saying that they have been there since this morning. Others when they arrive late are not attended to anymore and are told to come the next day”* (**health worker with 25 years of experience**);

The late arrival of pregnant mothers suggested by health workers is further supported by a mother making the following comment:

*“Nothing, I get there* (in the prenatal clinic) *when they have finished the teaching, so I don’t get anything. I don’t know what they teach. I don’t get any teaching***(adult mother)**

In addition, some health workers thought that some mothers deliberately arrived late for the antenatal session in order to avoid the long waiting time and the initial talks that were perceived to be boring and monotonous.

*“It is also possible that these people* (pregnant mothers) *come late because they want to come when the health education part is finished”***(health worker with 26 years of experience)**

While prenatal care is organised on a daily basis, the health education component is not routinely offered to pregnant women, but only under the form of ad hoc talks when time was available. The peripheral and low value of health education in prenatal sessions was confirmed by this health worker:

*“We don’t give health education talks every day, it is organised at least twice in a week so you can see that just vital things are actually left out…the good part is that when health education is not done properly it is not dangerous in the end, it may not contribute to somebody’s death (laughter*) (***health worker with 11 years of experience*)**

Almost all women indicated that the topics covered during the prenatal sessions included Malaria, HIV and immunization for which national programmes already exist. In addition, these sessions focused on the message to deliver at the hospital or a health centre with particular attention to what to bring with them at the time of delivery. The disease specific information that prenatal mothers received from the health workers on malaria and HIV/AIDS illustrated that these diseases received more attention compared to more important aspects of prenatal care as was illustrated by this mother:

*“No, they don’t teach us anything concerning feeding, what to do during pregnancy or even how to look after the baby. They only tell me that I am negative* (*that is HIV). That is what they normally tell me”* (**adult mother**)

Mothers most frequently received postnatal information from health workers on childhood immunization; they were advised to do so before they were discharged after delivery.

*“At the hospital once you deliver, there is nothing else that they tell you. No, they don’t tell us to go back. They only tell us to take the baby for immunization after delivery that’s it”* (**adult mother**), *“Once the baby is out, the rest is your business. They do not tell anything else. And I don’t think they have that time. What are they supposed to tell us? They tell us to take children for immunizations”* (**adult mother**).

This assertion was supported by responses from the health workers. In addition to this information and the distribution of a mosquito net, ‘*mama kits’* (containing disposable materials necessary for conducting a normal delivery) were also distributed.

*“When they come here we talk to them about HIV/AIDS, to sleep under a mosquito net…they even get free nets those who are coming for the first time, these days we also have mama kits from NMS* [National Medical Stores which is the central national store responsible for procurement, storage and distribution of essential medicines] *”* (**health worker 29 years of experience**)

During the prenatal sessions, health workers advised mothers to acquire the necessary requirements for delivery. Mothers were required to buy items such as surgical gloves, polythene papers (commonly referred to as *kaveera*) and to carry them along at the time of labour.

*“The health workers tell us to come with razorblade, basin, gloves, kaveera and thread. I even bought the medicine that stops bleeding* (meaning ergometrine)*, needle and syringes”***(adolescent mother)**

#### Mothers as passive recipients of prenatal services

Health workers did not consider mothers as active recipients of antenatal care. They rather approached them as passive recipients, largely ignoring mothers’ views regarding pregnancy, delivery and neonatal care. Health education sessions were basically a one-way process in which health workers assumed they know the mothers’ needs and they should simply conform to what is being told. A health worker reported:

*“because health education is where all mothers are supposed to listen and pick things, what they are told,…so people don’t take things* [the educational information offered] *seriously, but they get the information”*(**health worker 26 years of experience**)

Mothers were not informed on the results of laboratory tests such as haemoglobin/anaemia analysis. At the time of delivery mothers were never updated on the progress of labour, for example on the descent of the presenting part. Women were worried that nurses would treat patients without explaining which procedures they were about to perform. One mother, still indignant, had this to say:

“*Ha, I was in the hospital, the nurse came and injected a patient without even touching her. It was like she was injecting a cow”* (**adult mother**)

#### Source of prenatal information

For education on pregnancy and newborn care, family networks were a prominent source of information among mothers, while none of them mentioned health workers as an information source. During prenatal visits to the health centre or hospital, no specific advice was offered on maternal nutrition.

*“Nothing, I never heard anything like that* (teaching on caring for the newborn baby)*. Perhaps they teach other mothers what to eat but me I never heard anything. Nothing about how to nurse the baby or feed the baby. Nothing!”* (**adult mother)**

Hence, practices depended on individual decisions and preferences largely influenced by advice obtained from family members, relatives and peers. Unfortunately, this advice was not based on any scientific evidence, but rather on myths and traditional cultural beliefs. Both adolescent and adult lactating women expressed similar sentiments:

*“When it was almost time to deliver, I was told by my mother-in law to sit on the potato leaves so that I could soften and dilate easily the birth canal”* (**adolescent mother**)

*“When it comes to breastfeeding I would observe the wives of my husband’s brothers and my brother’s wife so I could see what to do, I just learnt by myself* (laughter)*”***(adult mother)**

In particular, mothers were concerned about foods that might make the baby in the womb grow too big which may lead to a surgery and the chance of dying during this procedure was believed to be very high. In addition to the advice about certain foods, women received the recommendation from their close relatives to abstain from sexual intercourse as it was believed to cause a white cheesy coating (vernix caseosa) on the skin of the newborn. As such some mothers had this to say:

*“It depends on how you feel. Some people fear maize porridge, it makes the baby too big, they* (relatives) *tell us to eat green vegetables, not to eat things that make the baby too big, that when the baby becomes big, it will be hard to deliver the baby and then you can get operated or die”***(adolescent mother)**

*“I was told by my mother that I should stop* (having sexual intercourse) *when I was seven months pregnant, that when you sleep with a man in late pregnancy you will deliver a baby which is dirty with a bad skin”* (***adolescent mother***)

Similarly, the care and advises regarding newborn care offered in the health centre and at the hospital after delivery, were perceived to be poor. These were some of the comments from adolescent women:

*“Ooh oh, can the nurse at Kiryandongo touch your baby. Their job is to get the baby out of your womb. Once that is done, they are finished. They do not tell you when to breastfeed, when to bathe, nothing Eeh! Nothing! Do you think those nurses tell you anything? Nothing, their job is to help you deliver and that’s it, nurses should be like mothers who know what childbirth means!”*(**adolescent mother**)

These concerns about the baby having vernix caseosa at birth, a big-size baby and fear of being operated were reported among both adolescent and adult mothers. However the prenatal intake of *waragi* (*potent gin, locally brewed from ripe bananas*)*,* which was believed to help in cleansing the baby’s cheesy skin while in the womb, was only reported among adolescent mothers:

*“Yes I was taking a little waragi; I took about a full glass once a week. I started when I got pregnant. I was told that a little waragi is good for the baby, it helps the skin of the baby to remain clean”* (**adolescent mother**)

### Newborn care practices: mothers’ perceptions

Data on current newborn care practices is presented along three specific thematic areas: cord care, thermal care and initiation of breastfeeding.

*Cord care:* The majority of mothers used strings from old household materials, such as sacks for tying the cord. Razorblades and other sharp objects available at home, were mostly used for cutting the cord. Only a few mothers used newly bought strings and razorblades for tying and cutting the cord. All interviewed mothers applied substances to the cord to facilitate the healing process. Some applied ashes which were obtained from burning specific shrubs whilst others applied baby powder, cooking oil, local herbal substances and animal dung.

*“We use thread from the sacks* [sacks of sugar]*”* (**adolescent mother**), *“I used reeds. The dry reeds are very sharp and they can cut. I have used these since my first born. That is what we use”* (**adult mother**)

Some mothers primarily applied substances like salty water and baby powder, but reported that in case these did not have the desired effect, they chose other options as suggested by this mother:

*“I used powder for about three days but it didn’t help, then I asked people and they told me ekiyondo* (*locally mixed herb*) *that’s what I used and the cord got dry”* (**adult mother**)

#### Thermal care

Mothers generally used bed sheets or their own clothes for the provision of warmth immediately after delivery, while some mothers used *bitenge* (piece of cotton cloth measuring about two-by-two and a half metre, usually wrapped around their waists as part of their dress cord). After delivery, mothers shared the same bed and blanket with their baby while sleeping. Almost all bathed their newborn babies immediately or within 24 hrs after giving birth. Some mothers bathed their baby in the cold night.

*“I bathe the baby immediately. Once I finished cleaning myself, I put some water on fire and when it was ready, I bathe the baby. Even if it is night, I always bathe the babies immediately”* (**adult mother**);

This practice of immediate bathing was the same for those who delivered in a health facility or at home. Those mothers who delivered unassisted at home, bathed their babies earlier than those who delivered at the health facility, were assisted by Traditional Birth Attendants (TBA) or by relatives.

A dirty skin at birth had a strong bearing on how soon the baby will be bathed. If the baby was born with the vernix caseosa, then he/she was bathed immediately, while bathing of a ‘clean’ baby could be delayed by a few hours:

*“When the baby is born dirty* (*with the white coating*) *we bathe immediately. Some children are born when they’re clean, but when they are dirty we have to bathe immediately, because a visitor who comes to see the baby cannot carry a dirty baby”* (***adult mother***)*.*

A delay in bathing the baby was found among adolescent mothers. This decision was driven by fear and inexperience rather than based on evidence as suggested by an adolescent mother:

*“The baby was bathed the next day when we got home I did not know how to do it (*bathing*). I also never wanted to look at the cord. It is scary”* (**adolescent mother**)

#### Initiation of breastfeeding and pre-lacteal feeds

Generally, early crying of the baby prompted women to initiate breastfeeding early:

*“You wait; bathe the baby and when the baby cries then you breastfeed”* (**adult mother**), *“I normally breastfeed once the baby cries. If it does not cry, I breastfeed the next day”* (**adult mother**)

However, initiation of breastfeeding was often delayed for a few hours up to two days due to the fact that the baby was sleeping or there was no let-down of breast milk yet.

*“I delivered at about 6.00 p.m. and he was breastfed the next day in the evening”* (**adolescent mother**)*, “I breastfeed after the breast milk has come this can be one or two days after”* (**adult mother**)

Before initiating breastfeeding, women gave pre-lacteal drinks to the baby such as plain boiled water, boiled water with sugar or with glucose or some tea without sugar. Nonetheless, very few mothers did not provide pre-lacteal feeds but waited for the let-down of breast milk.

*“I didn’t give the baby anything until the breast milk came. I just kept on putting him (*the baby*) on the breast”***(adult mother),**

There was a difference in preference for the type of pre-lacteal feeds offered by the two age-groups of mothers when breastfeeding was not initiated. The adolescent mothers appeared to have a preference for a combination of water and glucose, whilst adult mothers seemed to have a preference for water, sugar, salt or tea:

*“We give glucose. We put it in water and give it to the baby with a spoon”***(adolescent mother),***“There was no breast milk so we gave the baby glucose”***(adolescent mother)**

*“When there’s no breast milk, I boil water and add sugar then I give it to her with a spoon”***(adult mother),***“I boil water and add sugar and salt then give it to the baby for about 2 days. In two days the breast milk comes”***(adult mother)**

### Insights from trainers and employers regarding newborn care

According to the trainers at the nurse training school, enrolled and registered nurses/midwives have similar curriculums regarding prenatal and newborn care; but important differences exist. Enrolled nurses/midwives together with the registered nurses mainly received a theoretically oriented training with minimal practical sessions while registered midwives in addition to their training regarding domiciliary care, devote two-thirds of their training to practical interactions with the mothers and babies. Domiciliary training is mandatory and prerequisite for sitting hospital final exams and receiving a certificate of registration as a practising midwife. After completion of domiciliary training a trainee midwife is issued with a certificate of successful completion:

*“Domiciliary care is a compulsory training for all registered midwives. During domiciliary care a trainee midwife should be able to confirm established labour; manage all the four stages of labour; after delivery, assess the mother for discharge; and follow-up the mother in her home for seven days after birth”***(tutor training school).**

*“Most participants enrolled for the basic midwifery training are young high school graduates with no prior experience in newborn care, whereas students of the advanced training in midwifery did, and yet they (enrolled midwives) are expected to provide all the required information and treatment to pregnant mothers ”***(tutor training school)***.*

The employers at the district noted that the Ministry of health together with the Ministry of public service publish job specifications, structures and job requirements for various Ministries of Health (MOH) institutions including health centres at the district. These documents spell out job titles, key outputs and activities related to the job, and person’s specifications. This is what one employer had to say:

*“These guidelines are centrally formulated for strict and rigid implementation at the district level. The ministry of public service ensures its strict implementation by scrutinizing staffs’ monthly payrolls and any irregular deployment will lead to suspension of such a payment. Rigid deployment rules do not allow individual health managers of the district to exercise flexibility in the deployment process. For example a District Health Officer cannot deploy registered midwives to a health centre of level 3 even if assessment of the local need requires the presence of such a highly qualified midwife”* (**employer at the district**)*.*

In regard to the inadequate information offered at the prenatal clinics, employers at the district suggested that the few health workers could not attend the high number of patients who are seeking care, hence increasing the workload:

*“Apart from antenatal care being labour-intensive, the number of health workers are few compared to the number of mothers who come for antenatal care. Health workers cannot give all the necessary information required during antenatal. They can skip other activities in order to try and serve all the mothers who have reported to the health centre” (****employer at the district****)*

Tutors at the training school referred to the lack of adequate training for enrolled nurses/midwives and registered nurses to offer the entire information package. Instead, the scope of work designated for enrolled nurses/midwives and registered/nurses are limited to conducting a normal delivery (for example a cephalic presentation, delivering a mother who is having her second or third baby) but not attending to a complicated labour like a breach presentation or removal of a retained placenta. Moreover enrolled nurses/midwives and registered nurses do not get training in domiciliary care, as suggested by one tutor:

*“That is not what they* (enrolled nurses/midwives and registered nurses) *were trained to do (*providing information on newborn care*). They were trained to manage normal labour and recognise any complication and be able to refer to the next level of care or where there is a registered midwife or doctor. That is the reason why enrolled midwives or nurses do not waste time in giving detailed information to prenatal or immediate postnatal mothers”***(tutor in training school)**

## Discussion

Our findings highlight important problems in the organisation of prenatal care in the health care system in Masindi. Even though the interviewed mothers made on average 3.2 prenatal care visits during their pregnancy, they were inadequately prepared to provide recommended newborn care, suggesting serious flaws in the current organisation and offer of prenatal services in the health care centres. The focus of the prenatal care tended to be selectively oriented towards the management of health problems for which vertical programmes already exist. In addition, communication and attitudinal problems between health workers and pregnant mothers were present. More so, the current training curriculum and guideline for allocation of midwives and nurses to health centres impede the adequate offer of educational information regarding newborn care to pregnant mothers in the rural areas.

### Current newborn care practices

Our results showed that women commonly used unhygienic materials for tying and cutting the cord; bathed babies soon after delivery and; delayed initiation of breastfeeding while offering pre-lacteal feeds; predisposing their newborns to early infections and hypothermia. This is an important finding, in light of the fact that pregnant mothers made sufficient contact with the health care system during the prenatal period; suggesting that vital educational interventions about thermal care, initiation of breastfeeding and clean cord care practices were not discussed carefully with the mothers. Yet, this moment could be optimally utilised by health workers to offer relevant information and engage with pregnant mothers to discuss important matters related to pregnancy and childbirth [26].

Our findings are similar to newborn care practices reported in eastern Uganda where less than half of the mothers were found to practice optimal newborn care [27] and were unwilling to accept delayed bathing or leaving the cord to dry without application of substance [28]. Similar studies on newborn care practices from rural India indicate that initiation of breastfeeding was delayed up to three days after birth when it is believed that the let-down of breast milk occurred [29]. In Ghana, contrary to our findings, clean delivery practices (hand-washing, delivery on a clean surface, use of sterile material for tying and cutting the cord) were widely practiced even among home deliveries [30]. This was possible in Ghana because a community intervention to improve newborn care practices invested in understanding community behaviours through formative studies and adapted recommended interventions to suit local contexts [31]. These findings indicate that the design and implementation of targeted community interventions can be promising to improve newborn care practices in Uganda. Moreover, the Ghana study found that health workers perpetrated wrong practices hence additional training and monitoring for health care providers was implemented as a strategy to improve newborn care practices [31].

### Focus on vertical health programmes

Information offered to pregnant mothers was narrow in scope and focused mainly on malaria, HIV/AIDS and immunization in children. This is an important finding as for these diseases vertical programmes are in place in Uganda. WHO proposed the integration of vertical programmes such as the prevention of malaria and HIV/AIDS into prenatal care [32]. However, the challenge remains to arrive at an optimum between general health services and these vertical programmes, since most of the offered information was not aligned to the real concerns expressed by prenatal mothers. There are significant inflows of resources for specific disease control projects that are often implemented in a vertical way. Previous research has shown that these programmes often utilise prenatal care services as a portal of entry into the health care system, bypassing existing administrative and operational structures of the general health system [33]. This double agenda may partly explain why mothers received information on HIV/AIDS, malaria and immunization and much less on recommended newborn care practices. Our findings were similar to studies conducted in eastern Uganda and southern Tanzania and suggest that health workers did not allocate sufficient time to discuss important issues regarding pregnancy and newborn care [34,35]. Vertical programmes often offer in-service training for health workers; they provide financial incentives and often superior infrastructure compared to the general health system [36]. These could negatively incentivise health workers to pay more attention to such programmes and produce unintended consequences like diversion of attention hence undermining the broader horizontal prenatal health services.

### Attitudinal and communication problems

Mothers who made antenatal care visits were treated as passive recipients of antenatal care, which is contrary to the widely acknowledged patient-centredness model emphasising an active and autonomous patient [37]. Health workers viewed mothers as individuals without considering their pregnancy and later on the newborn baby. Information during prenatal sessions is provided as instructions which must be adhered to and this might have been intimidating for the pregnant women and their families. This approach lends itself to two fundamental flaws - first that mothers do not know what to do and therefore the health workers’ role is to direct her on what is right, ignoring the fact that most mothers have a preconceived mind about possible care practices [38]; secondly, it assumes that all mothers attending antenatal sessions are a homogenous group demanding the same information [39]. As stated previously this could have been counter-productive for behaviour change and improved newborn care practices [38]. However, educational interventions during prenatal sessions require communication skills and sufficient time dedicated for dialogue on the part of the caregiver [5,40].

Unfortunately, information offered during subsequent antenatal education sessions pointed to a gross lack in continuity of (preventive) care. These findings are consistent with the findings by Conrad and others [35] whereby the different health education sessions did not take into consideration previous education topics, nor the mothers’ knowledge, leading to possible duplication and monotony of information [41]; further aggravated by the lack of individualised health education [35], also highlighted in our study.

To achieve full potentials of prenatal services, the WHO recommends four visits offering essential evidence-based interventions during pregnancy relying on the individual mothers’ needs to make decisions bearing in mind the uniqueness of each pregnancy [32,42]. These recommendations suggest the systematic offer of intervention packages relevant to a particular gestation age; meaning that pregnant mothers progressively get better prepared to care for their pregnancy and the newborn baby.

### Sources of information

Since mothers were bombarded with general information during prenatal health education sessions assuming they will learn what suits them, immediate relatives became by default an important alternative source of information regarding pregnancy and newborn care. Moyer et.al described relatives as critical social gatekeepers having extremely powerful social positions [43]. The information that mothers obtained, mainly from immediate relatives is rooted in tradition without necessarily a firm scientific evidence base [44]. Relatives often play leading roles during the prenatal, intra-natal, and immediate postnatal period; are extremely powerful influencing the frequency of bathing and breastfeeding of the baby and application of substance on the cord. However, in principle, relatives should complement sound and patient-centred care offered by professionals and not to replace it. In Ghana, the problem of relatives offering the wrong type of information was bypassed initially by conducting formative studies and later by actively engaging various stakeholders from the inception throughout the intervention in order to affect behaviour change for newborn care [31,45,46]. Similar engagement of district officials should be tried in Uganda.

#### Deficiency in the health system

Our data highlights one important overarching problem underlying all four aforementioned problems, i.e. guidelines for training and deployment of nurses and midwives. The Ugandan health system recommends deployment of enrolled nurses and enrolled midwives to health care of level 2 and 3, yet the curriculum of the enrolled midwives and enrolled nurses does not prepare them to inform and support the mothers. Although enrolled nurses/midwives together with registered nurses/midwives are expected to offer equal prenatal and immediate postnatal information to mothers, their training is different. This suggests that different prenatal and immediate postnatal information should be offered at the several health centre levels. In this study however, we did not examine the difference in scope of provided prenatal and immediate postnatal information between the tiers of care. Further investigation into the knowledge and practice of all those who are in contact with mothers similar to what was examined in Ghana [31] is suggested.

### Strengths and limitations

This study collected data from health service users health service providers as well as employers and trainers of health workers hence examining the situation of newborn care from a broader perspective [26]. Since pregnant mothers were visited in their homes information was obtained from both mothers who utilised prenatal services as well as from those who never made contact with the health care system during pregnancy. The study involved two districts with a diverse ethnicity unlike similar studies conducted among a homogenous community in Eastern Uganda [27]. This study employed a qualitative approach of data collection therefore able to explore in-depth of newborn care practices and provide plausible explanations for such observed behaviours. This was further strengthened by application of several aspects of qualitative techniques [29]. Moreover a triangulation approach to data analysis was used in order to increase the validity of our results. Interviews were conducted among women with children, implying that those who lost their children before the interview were never included and therefore their story was not documented in this study. This study was limited to the offer of prenatal information and newborn care practices. It is however desirable to explore social and cultural influences of communities on newborn care practices. Finally, we did not specify in this study at which health centre levels mothers made their different antenatal consultation. Consequently we cannot draw conclusions on whether hospital or health centres workers offered different prenatal or immediate postnatal services.

## Conclusions

This study highlights important problems with organization and offer of prenatal care services in the health centres which leaves pregnant women inadequately prepared for their pregnancy and newborn care. There is no personalised information; health education is practised from the perspective of information transfer in a top-down way and health workers are not sufficiently trained to support and prepare the pregnant women to be capable mothers. Prenatal care should be realigned to integrate the educational component of newborn care, based on the principles of patient-centred care and accept individual mothers as active participants. Moreover there is a differential training curriculum and deployment criteria for nurses and midwives yet they are meant to offer an equal package of prenatal and immediate postnatal care education, which calls for further exploration of their training curriculum.

The findings of this study regarding newborn care practices should be used to develop community interventions that can encourage recommended newborn care practices.

## Competing interests

The authors declare that there is no competing interest.

## Authors’ contributions

RMA, BC, LA, CGO and PK conceptualised and designed the study, RMA collected and transcribed the data; RMA, KVR, RV and PK analysed the data; RMA, KVR, RV wrote the manuscript; BC, CGO and PK coordinated and provided technical support throughout the process of developing this study; all authors read and approved the final manuscript.

## Pre-publication history

The pre-publication history for this paper can be accessed here:

http://www.biomedcentral.com/1471-2393/13/176/prepub

## References

[B1] Black RE Cousens S Johnson HL Lawn JE Rudan I Bassani DG Jha P Campbell H Walker CF Cibulskis R Global, regional, and national causes of child mortality in 2008: a systematic analysis Lancet 2010 375 9730 1969 1987 20466419 10.1016/S0140-6736(10)60549-1

[B2] MOH Save the Children, UNICEF, WHO Situation Analysis of Newborn health in Uganda: Current status and opportunities to improve care and survival 2008 Kampala: Government of Uganda

[B3] Lawn JE Cousens S Zupan J 4 million neonatal deaths: when? Where? Why? Lancet 2005 365 9462 891 900 15752534 10.1016/S0140-6736(05)71048-5

[B4] Lawn J Shibuya K Stein C No cry at birth: global estimates of intrapartum stillbirths and intrapartum-related neonatal deaths Bull World Health Organ 2005 83 6 409 417 15976891 PMC2626256

[B5] WHO Standards for Maternal and neonatal care development2002a Geneva: World Health Organisation Department of Making pregnancy safer

[B6] Darmstadt GL Bhutta ZA Cousens S Adam T Walker N de Bernis L Evidence-based, cost-effective interventions: how many newborn babies can we save? Lancet 2005 365 9463 977 988 15767001 10.1016/S0140-6736(05)71088-6

[B7] Kumar V Mohanty S Kumar A Misra RP Santosham M Awasthi S Baqui AH Singh P Singh V Ahuja RC Effect of community-based behaviour change management on neonatal mortality in Shivgarh, Uttar Pradesh, India: a cluster-randomised controlled trial Lancet 2008 372 9644 1151 1162 18926277 10.1016/S0140-6736(08)61483-X

[B8] WHO Integrated management of pregnancy and childbirth: Managing newborn problems; a guide for doctors, nurses and midwives 2003 Geneva: Department of Reproductive Health and Research, World Health Organisation

[B9] WHO Mother-Baby package: Implementing safe motherhood in countries. Practical guide 1994 Geneva: Division of Family Health World Health Organisation Maternal Health and Safe Motherhood Pragramme

[B10] Kinzie B Gomez P Basic Maternal and Newborn Care: A guide for skilled providers 2004 Baltimore JHPIEGO/MNH program

[B11] WHO Making every mother and child count The World Health Report 2005 2005 Geneva: World Health Organisation

[B12] Guliani H Sepehri A Serieux J What impact does contact with the prenatal care system have on women’s use of facility delivery? Evidence from low-income countries Soc Sci Med 2012 74 12 1882 1890 22483706 10.1016/j.socscimed.2012.02.008

[B13] De Allegri M Ridde V Louis VR Sarker M Tiendrebeogo J Ye M Muller O Jahn A Determinants of utilisation of maternal care services after the reduction of user fees: a case study from rural Burkina Faso Health Policy 2011 99 3 210 218 21056505 10.1016/j.healthpol.2010.10.010

[B14] WHO Working with Individuals, Families and Communities to improve Maternal and newborn health Bulletin of the World Health Organisation, department of Making Pregnancy Safer 2010 Geneva: World Health Organisation WHO/MPS/09.04

[B15] Rahman M Haque SE Zahan S Islam O Noninstitutional births and newborn care practices among adolescent mothers in Bangladesh J Obstet Gynecol Neonatal Nurs 2011 40 3 262 273 10.1111/j.1552-6909.2011.01240.x 21585526

[B16] Van Eijk AM Hanneke MB Odhiambo F Ayisi JG Blokland IE Rosen DH Adazu K Slutsker L Lindblade KA Use of antenatal services and delivery care among women in rural western Kenya: a community based survey BMC: Reprod Health 2006 3 2 10.1186/1742-4755-3-2 PMC1459114 16597344

[B17] Kabali E Gourbin C De Brouwere V Complications of childbirth and maternal deaths in Kinshasa hospitals: testimonies from women and their families BMC Pregnancy Childbirth 2011 11 29 21496262 10.1186/1471-2393-11-29 PMC3095568

[B18] UN Millenium Development Goals Report 2010 2010 New York: United Nations We can end Poverty 2015 MDGs

[B19] UDHS Uganda Demographic and Health Survey 2006 Kampala Uganda: Uganda Bureau of Statistics Government of Uganda

[B20] Tann CJ Kizza M Morison L Mabey D Muwanga M Grosskurth H Elliott AM Use of antenatal services and delivery care in Entebbe, Uganda: a community survey BMC pregnancy and childbirth 2007 7 23 17931422 10.1186/1471-2393-7-23 PMC2098779

[B21] Oestergaard MZ Inoue M Yoshida S Mahanani WR Gore FM Cousens S Lawn JE Mathers CD Neonatal mortality levels for 193 countries in 2009 with trends since 1990: a systematic analysis of progress, projections, and priorities PLoS Med 2011 8 8 e1001080 21918640 10.1371/journal.pmed.1001080 PMC3168874

[B22] Waiswa P Understanding Newborn Care in Uganda-Towards Future Interventions. Thesis for doctoral degree (PhD) 2010 Stockholm Sweden: Department of Health Policy, Planning and Managament, Makerere University, School of Public Health, College of Health Sciences, Kampala Uganda and the division of Global Health (IHCAR) Department of Public Health Sciences, Karolinska Institutet

[B23] Byaruhanga RN Nsungwa-Sabiiti J Kiguli J Balyeku A Nsabagasani X Stefan P Hurdles and opportunities for newborn care in rural Uganda Midwifery 2011 6 27 775 80 20685016 10.1016/j.midw.2010.02.005

[B24] Aniebue UU Aniebue PN Women’s perception as a barrier to focused antenatal care in Nigeria: the issue of fewer antenatal visits Health Policy Plan 2010 26 5 423 428 21088079 10.1093/heapol/czq073

[B25] QSR NVivo 8 Fundamentals QSR International pty Ltd 2008 Australia: Doncaster, Victoria 3108 ABN 47 006 357 213(Version 8)

[B26] Sword W Heaman MI Brooks S Tough S Janssen PA Young D Kingston D Helewa ME Akhtar-Danesh N Hutton E Women’s and care providers’ perspectives of quality prenatal care: a qualitative descriptive study BMC pregnancy and childbirth 2012 12 29 22502640 10.1186/1471-2393-12-29 PMC3352181

[B27] Waiswa P Peterson S Tomson G Pariyo GW Poor newborn care practices - a population based survey in eastern Uganda BMC Pregnancy Childbirth 2010 10 9 20178626 10.1186/1471-2393-10-9 PMC2834614

[B28] Waiswa P Kemigisa M Kiguli J Naikoba S Pariyo GW Peterson S Acceptability of evidence-based neonatal care practices in rural Uganda - implications for programming BMC Pregnancy Childbirth 2008 8 21 18570672 10.1186/1471-2393-8-21 PMC2459143

[B29] Iyengar SD Iyengar K Martines JC Dashora K Deora KK Childbirth practices in rural Rajasthan, India: implications for neonatal health and survival J Perinatol 2008 28 Suppl 2 S23 S30 19057565 10.1038/jp.2008.174

[B30] Hill Z Tawiah-Agyemang C Okeyere E Manu A Fenty J Kirkwood B Improving hygiene in home deliveries in rural Ghana: how to build on current attitudes and practices Pediatr Infect Dis J 2010 29 11 1004 1008 20811311 10.1097/INF.0b013e3181f5ddb1

[B31] Hill Z Manu A Tawiah-Agyemang C Gyan T Turner K Weobong B Ten Asbroek AH Kirkwood BR How did formative research inform the development of a home-based neonatal care intervention in rural Ghana? J Perinatol 2008 28 Suppl 2 S38 S45 19057567 10.1038/jp.2008.172

[B32] WHO Ornella L, Seipati M-A, Patricia G, Munjanja S Antenatal Care Opportunities for Africa's Newborns 2006 Geneva: World Health Organisation

[B33] Horton R What will it take to stop maternal deaths? Lancet 2009 374 9699 1400 1402 19783292 10.1016/S0140-6736(09)61669-X

[B34] Sarker M Schmid G Larsson E Kirenga S De Allegri M Neuhann F Mbunda T Lekule I Müller O Quality of antenatal care in rural southern Tanzania: a reality check BMC Res Notes 2010 3 209 10.1186/1756-0500-3-209 PMC3161364 20663202

[B35] Conrad P De Allegri M Moses A Larsson EC Neuhann F Muller O Sarker M Antenatal care services in rural Uganda: missed opportunities for good-quality care Qual Health Res 2012 22 5 619 629 22232296 10.1177/1049732311431897

[B36] Pfeiffer J Montoya P Baptista AJ Karagianis M Pugas Mde M Micek M Johnson W Sherr K Gimbel S Baird S Integration of HIV/AIDS services into African primary health care: lessons learned for health system strengthening in Mozambique - a case study J Int AIDS Soc 2010 13 3 20180975 10.1186/1758-2652-13-3 PMC2828398

[B37] Mead N Bower P Hann M The impact of general practitioners’ patient-centredness on patients’ post-consultation satisfaction and enablement Soc Sci Med 2002 55 2 283 299 12144142 10.1016/s0277-9536(01)00171-x

[B38] Vishwajeet K Aarti K Darmstadt GL Behavior change for newborn survival in resource-poor community settings: bridging the gap between evidence and impact Semin Perinatol 2010 34 6 446 461 21094419 10.1053/j.semperi.2010.09.006

[B39] Chalmers B How often must we ask for sensitive care before we get it? Birth 2002 29 2 79 82 12000410 10.1046/j.1523-536x.2002.00148.x

[B40] WHO WHO Antenatal care randomised trial: Manual for the implementation of the new model2002b Geneva: World Health Organisation Department of Reproductive Health and research family and community health

[B41] Conrad P Schmid G Tientrebeogo J Moses A Kirenga S Neuhann F Muller O Sarker M Compliance with focused antenatal care services: do health workers in rural Burkina Faso, Uganda and Tanzania perform all ANC procedures? Trop Med Int Health 2012 17 3 300 7 22151853 10.1111/j.1365-3156.2011.02923.x

[B42] WHO FOCUSED ANTENATAL CARE: Providing integrated, individualised care during pregnancy Geneva: World Health Organisation http://www.accesstohealth.org/toolres/pdfs/accesstechbrief_fanc.pdf, accessed 9th January 2013

[B43] Moyer CA Aborigo RA Logonia G Affah G Rominski S Adongo PB Williams J Hodgson A Engmann CM Clean delivery practices in rural northern Ghana: a qualitative study of community and provider knowledge, attitudes, and beliefs systems BMC pregnancy and childbirth 2012 12 1 50 22703032 10.1186/1471-2393-12-50 PMC3482570

[B44] Yakoob MY Menezes EV Soomro T Haws RA Darmstadt GL Bhutta ZA Reducing stillbirths: behavioural and nutritional interventions before and during pregnancy BMC Pregnancy Childbirth 2009 9 Suppl 1 S3 19426466 10.1186/1471-2393-9-S1-S3 PMC2679409

[B45] Kirkwood BR Manu A Tawiah-Agyemang C ten Asbroek G Gyan T Weobong B Lewandowski RE Soremekun S Danso S Pitt C NEWHINTS cluster randomised trial to evaluate the impact on neonatal mortality in rural Ghana of routine home visits to provide a package of essential newborn care interventions in the third trimester of pregnancy and the first week of life: trial protocol Trials 2010 11 58 20478070 10.1186/1745-6215-11-58 PMC2890650

[B46] Howe LD Manu A Tawiah-Agyemang C Kirkwood BR Hill Z Developing a community-based neonatal care intervention: a health facility assessment to inform intervention design Paediatr Perinat Epidemiol 2011 25 2 192 200 21281331 10.1111/j.1365-3016.2010.01178.x

